# Downregulation of testicular function in the goat by altrenogest

**DOI:** 10.1186/s12917-021-02845-6

**Published:** 2021-05-04

**Authors:** Lisa Mihsler-Kirsch, Henrik Wagner, Klaus Failing, Axel Wehrend

**Affiliations:** 1 Clinic of Obstetrics, Gynecology, and Andrology of Large and Small Animals with Ambulatory Service, Justus Liebig University Giessen, Frankfurter Strasse 106, 35392 Giessen, Germany; 2 AG Biomathematics and Data Processing, Justus Liebig University Giessen, Frankfurter Strasse 95, 35392 Giessen, Germany

**Keywords:** Altrenogest, Goat, Downregulation, Gonadal function, Castration

## Abstract

**Background:**

The present study investigated whether the administration of the progestin altrenogest provides noninvasive, temporary, and reversible suppression of gonadal function in the goat as a potential alternative to chirurgical castration, which is related with irreversibility, risks of complications till death of the animal and welfare issues. Eight sexually mature Peacock goats were randomly divided into two groups. The experimental group was administered altrenogest (0.088 mg/kg) orally once daily for 7 weeks. The remaining four goats received an oral glucose solution and served as the control group. After completing the administration period, the reversibility of the medication was evaluated for another 7 weeks (observation phase). The treatment effects were assessed by clinical examination; ultrasound examination of the testes, including one-dimensional grayscale analysis, blood testosterone levels, analysis of semen parameters and libido. At the end of the observation period, the animals were castrated and the testicles were examined histologically.

**Results:**

Altrenogest treatment had no significant effect on the physical development of the goats, the sonographic appearance of the testes, the gray values measured in the ultrasound images, or the blood testosterone levels. The effects of treatment on the testicular and semen parameters varied widely in the experimental animals; the testicle volume was significantly lower and the number of pathologically altered sperm in the ejaculate was significantly higher in treated animals.

**Conclusion:**

These findings indicate that daily altrenogest administration at a dose of 0.088 mg/kg does not reliably suppress gonadal function in the goat.

## Background

In contrast to other species, few studies have examined the effects of temporary suppression of gonadal function in the goat [1]. Today, the chirurgical castration is a widespread method to control reproduction and behavior in the male goat, but it is irreversible, covers the risk of complications till death of the animal and is a welfare issue because of the related pain. A temporary suppression of reproduction in bucks would make it possible to keep female and male animals together without breeding potentials, so that fertile bucks do not have to be kept in isolation until reproduction is desired again at a later time [1].

Previously evaluated methods of temporary gonadal function suppression in the goat include immunization against gonadotropin-releasing hormone [2–4], the use of long-term gonadotropin-releasing hormone implants [5], and administration of the α1-adrenoceptor antagonist tamsulosin [6].

In some species, altrenogest administration results in reversible downregulation of male reproductive function. Altrenogest is a synthetic progestogen which is widely used for estrus suppression and synchronization in pigs and mares by oral administration. The molecule binds to the progesterone receptors in the hypothalamus and pituitary gland resulting in inhibition of gonadotropins release [7].

In juvenile boars, daily administration of 20 mg altrenogest reduces plasma luteinizing hormone and testosterone levels, delays the onset of puberty, and reduces testicle size and weight [8, 9]. While daily altrenogest at 0.044 mg/kg has no significant effect on reproductive behavior and semen parameters in stallions and zebra stallions [10, 11], daily administration of 0.088 mg/kg altrenogest over a longer period to stallions significantly and reversibly reduces blood testosterone and luteinizing hormone concentrations, and induces a progressive decrease in testicle size and libido. Further, daily treatment with altrenogest at 0.088 mg/kg decreases the amount of ejaculate and the total sperm count in stallions throughout the treatment period [12, 13]. On the basis of these previous findings, the present study examined whether the administration of 0.088 mg/kg altrenogest has similar effects on the gonadal function in goats.

## Results

### Physical parameters, testicle measurements, and blood parameters

Clinical examination of the goats revealed no clear differences between the two groups. Body weight, length, and height increased during the course of the study (Table 1).

**Table 1:** Body parameters of bucks treated with altrenogest (AG, *n* = 4) vs. control animals (CG, n = 4) at seven time points. Results are given as mean ± standard deviation

	Body weight (kg)		Body lenght (cm)		Body height (cm)	
Time point	AG	CG	AG	CG	AG	CG
1	33.5 ± 3.8	35.4 ± 2.5	107.8 ± 3.2	107.8 ± 3.4	66.8 ± 4.6	67.3 ± 3.6
2	36.8 ± 3.9	38.0 ± 4.0	109.3 ± 4.0	110.0 ± 3.9	67.3 ± 5.0	67.8 ± 3.4
3	37.8 ± 5.6	39.2 ± 4.6	110.3 ± 3.7	110.3 ± 4.3	68.5 ± 2.7	68.3 ± 2.8
4	39.5 ± 6.5	40.7 ± 4.7	111.8 ± 3.3	112.3 ± 2.9	69.0 ± 2.6	69.0 ± 3.4
5	40.9 ± 6.7	41.8 ± 4.8	113.3 ± 2.2	114.0 ± 3.4	70.5 ± 3.1	70.4 ± 2.7
6	39.2 ± 6.8	41.3 ± 5.6	114.3 ± 0.5	116.3 ± 3.0	70.8 ± 4.2	71.8 ± 2.2
7	40.4 ± 6.4	43.8 ± 4.6	114.8 ± 0.8	117.8 ± 2.6	72.5 ± 4.3	72.0 ± 2.2

There was a highly significant effect of time, but no significant group difference (Table 2).

**Table 2:** Statistical analysis of physical parameters, testicular measurements, and testosterone concentrations of goats treated with altrenogest vs. control animals

Parameter	Factor		Interaction
	Group	Time	Time x Group
Body weight	0.64	< 0.0001	0.70
Body length	0.61	< 0.0001	0.63
Body height	0.93	< 0.0001	0.94
Volume of the testes	0.02	< 0.0001	0.0005
Consistency of the testes	0.14	0.04	0.07
Testosterone	0.65	0.026	0.59
Testes gray value	0.77	0.0002	0.11

Testes volume decreased in both groups during the experimental period so there was a highly significant effect of time. This reduction was more pronounced in the experimental group (Tables 2 and 3).

**Table 3:** Testes parameters of bucks treated with altrenogest (AG, *n* = 4) vs. control animals (CG, n = 4) at seven time points. Results are given as mean ± standard deviation. The consistency was categorized (see methods)

	Testes volume (cm^3^)		Testes consistency		Mean greyscale value	
Time point	AG	CG	AG	CG	AG	CG
1	101.5 ± 10.6	152.7 ± 31.9	1.0 ± 0.0	1.0 ± 0.0	18.0 ± 5.0	21.4 ± 2.2
2	54.9 ± 10.9	95.7 ± 28.4	2.0 ± 1.1	1.0 ± 0.0	19.5 ± 3.8	21.9 ± 3.0
3	48.8 ± 11.1	79.9 ± 28.5	2.0 ± 1.1	1.0 ± 0.0	20.6 ± 0.7	20.4 ± 3.9
4	45.9 ± 6.1	105.3 ± 21.3	2.5 ± 1.1	1.0 ± 0,0	23.0 ± 2.5	22.9 ± 1.8
5	52.6 ± 11.0	66.4 ± 15.1	2.2 ± 0.9	1.0 ± 0.0	20.1 ± 1.6	20.6 ± 3.5
6	67.3 ± 9.1	76.2 ± 16.1	1.7 ± 0.9	1.0 ± 0.0	23.0 ± 1.8	21.4 ± 3.2
7	66.4 ± 9.3	84.8 ± 16.0	1.2 ± 0.5	1.0 ± 0.0	24.5 ± 1.0	23.7 ± 2.0

Neither testosterone concentration nor the libido score did not differ significantly between groups (Tables 4 and 7).

**Table 4:** Libido and blood testosterone concentration of bucks treated with altrenogest (AG, *n* = 4) vs. control animals (CG, n = 4) at seven time points. Results are given as mean ± standard deviation. For categorization of libido see Table 8

	testosterone concentration (nmol / l)		libido sexualis	
Time point	AG	CG	AG	CG
1	1.2 ± 0.7	1.7 ± 0.5	3.7 ± 0.5	3.5 ± 0.5
2	2.1 ± 1.6	7.4 ± 12.4	2.0 ± 2.3	3.0 ± 1.1
3	4.2 ± 4.9	2.4 ± 1.9	2.5 ± 1.7	2.7 ± 1.8
4	11.3 ± 13.2	4.4 ± 3.9	2.5 ± 1.7	3.0 ± 0.8
5	5.0 ± 6.2	7.1 ± 6.0	2.2 ± 2.0	3.2 ± 0.9
6	8.8 ± 5.1	7.0 ± 5.6	1.7 ± 2.0	2.5 ± 1.9
7	16.0 ± 10.6	5.7 ± 5.6	3.0 ± 1.1	2.7 ± 1.8

### Semen parameters

Ejaculates could be obtained from all animals in the control group. It was only possible to collect semen from two bucks of the experimental group one time at the 7th time point, from the other two bucks in this group at any time. These two bucks showed normal ejaculate parameters (Tables 5 and 6).

**Table 5:** Volume, pH and sperm mass activity of ejaculates from bucks treated with altrenogest (AG, *n* = 4) vs. control animals (CG, n = 4) at seven time points. Results are given as mean ± standard deviation

	volume (ml)		pH		mass activity	
Time point	AG	CG	AG	CG	AG	CG
1	0.6 ± 0.2	0.6 ± 0.2	6.9 ± 0.4	6.6 ± 0.2	2.2 ± 0.5	2.0 ± 0.8
2	0.5 ± 0.4	0.4 ± 0.2	6.6 ± 0.1	6.7 ± 0.2	3.0 ± 0.0	2.2 ± 0.9
3	0.8 ± 0.2	0.3 ± 0.1	6.6 ± 0.2	6.8 ± 0.3	1.0 ± 1.4	3.0 ± 0.0
4	0.4 ± 0.0	0.4 ± 0.1	7.0 ± 0.2	6.7 ± 0.3	1.5 ± 2.1	1.7 ± 0.9
5	0.4 ± 0.4	0.3 ± 0.1	7.5 ± 0.7	6.9 ± 0.1	1.5 ± 2.1	2.0 ± 1.4
6	0.3 ± 0.2	0.3 ± 0.2	7.2 ± 0.0	7.0 ± 0.0	1.0 ± 1.4	2.3 ± 0.5
7	0.2 ± 0.2	0.3 ± 0.2	6.8 ± 0.2	6.9 ± 0.6	1.0 ± 1.4	1.3 ± 1.5

**Table 6:** Progressive sperm motility, sperm concentration and total sperm output of ejaculates from bucks treated with altrenogest (AG, *n* = 4) vs. control animals (CG, n = 4) at seven time points. Results are given as mean ± standard deviation

	progressive motility(%)		concentration (10^6^/ul)		total sperm output (10^12^)	
Time point	AG	CG	AG	CG	AG	CG
1	87.5 ± 15.0	73.7 ± 11.4	3.6 ± 1.8	2.5 ± 1.4	2.1 ± 1.1	1.6 ± 1.2
2	62.5 ± 13.5	71.2 ± 16.5	3.6 ± 0.3	1.2 ± 0.5	1.7 ± 1.3	0.5 ± 0.3
3	50.0 ± 28.2	70.0 ± 20.0	2.1 ± 0.7	1.1 ± 0.3	1.8 ± 1.2	0.3 ± 0.3
4	77.5 ± 10.6	67.5 ± 9.5	2.8 ± 0.7	1.3 ± 1.0	1.3 ± 0.2	0.6 ± 0.6
5	62.5 ± 31.8	81.2 ± 13.1	1.3 ± 0.1	1.3 ± 0.9	0.7 ± 0.9	0.5 ± 0.5
6	45.0 ± 49.5	70.0 ± 17.3	1.5 ± 0.8	1.7 ± 0.9	0.6 ± 0.6	0.6 ± 0.4
7	57.5 ± 38.9	55.0 ± 37.7	2.8 ± 1.7	2.4 ± 1.3	0.8 ± 0.0	1.0 ± 0.8

**Fig. 1:**
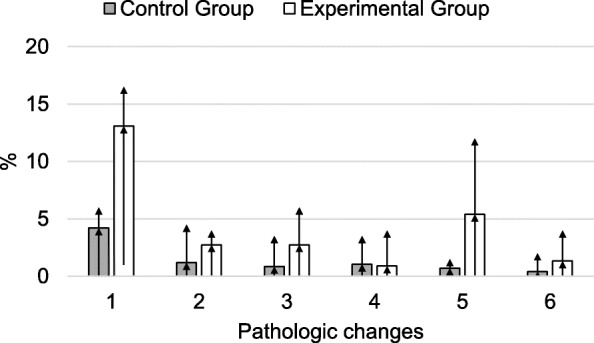
Pathologic changes in the ejaculate of the goats in percent, averaged over all the time-points, represented by the arithmetic mean values as well as the lowest and highest value (range). 1 = total percentage of pathologically changed sperm, 2 = detached heads, 3 = broken tails, 4 = looped tails, 5 = rolled tails, 6 = cytoplasmic droplets. Errors bars indicate the minimum and the maximum values

Time had a significant effect on the volume, density, and pH of the ejaculate, and a highly significant effect on the total sperm count (Table 7). The number of morphologically altered sperm was significantly higher in the experimental group (Fig. 1).

**Table 7:** Statistical analyses of semen parameters and libido score of altrenogest-treated vs. control goats

Parameter	Factor		Interaction
	Group	Time	Time x Group
Seminal volume	0.31	0.005	0.22
pH	0.33	0.03	0.13
Mass activity	0.57	0.93	0.10
Progressive motility	0.81	0.051	0.34
Concentration	0.14	0.006	0.25
Total sperm output	0.22	0.0004	0.10
Total defect	0.05	0.68	0.21
Libido score	0.11	0.12	0.21

### Ultrasound examination and gray value analysis

**Fig. 2:**
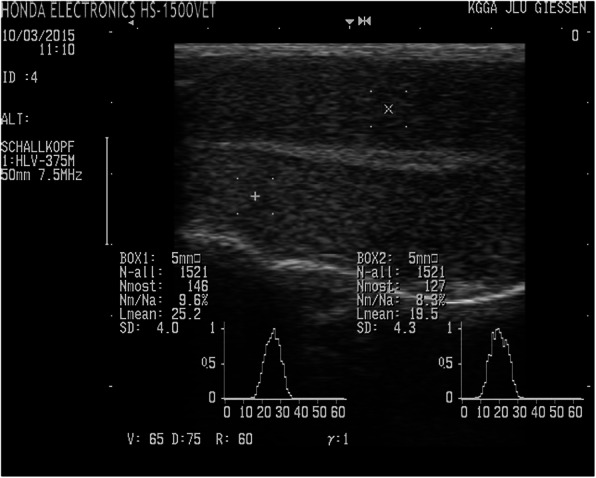
Longitudinal ultrasonography of a testis from a buck of the altrenogest group (HS 1500 V, Honda Electronics). Two regions of interest were marked to perform a grey-scale analysis of the parenchyma. 1: tunica vaginalis, 2: testicular parenchyma, 3: mediastinum

At all time-points, the testicular parenchyma presented as a homogeneous area with medium echogenicity, surrounded by a hyperechogenic area formed by the tunica vaginalis, the internal spermatic fascia, and the skin. The mediastinum could always be visualized and formed in the longitudinal section of the testicle as a narrow, homogeneous, centrally located strip or as a central point in the cross section of the testis and was more hyperechoic than the parenchyma (Fig. 2). The sonographic appearance of the testes did not differ significantly within or between animals at any time-point. The effect of time was highly significant, but there was no significant effect of treatment (Tables 2 and 3).

### Histologic examination

**Fig. 3:**
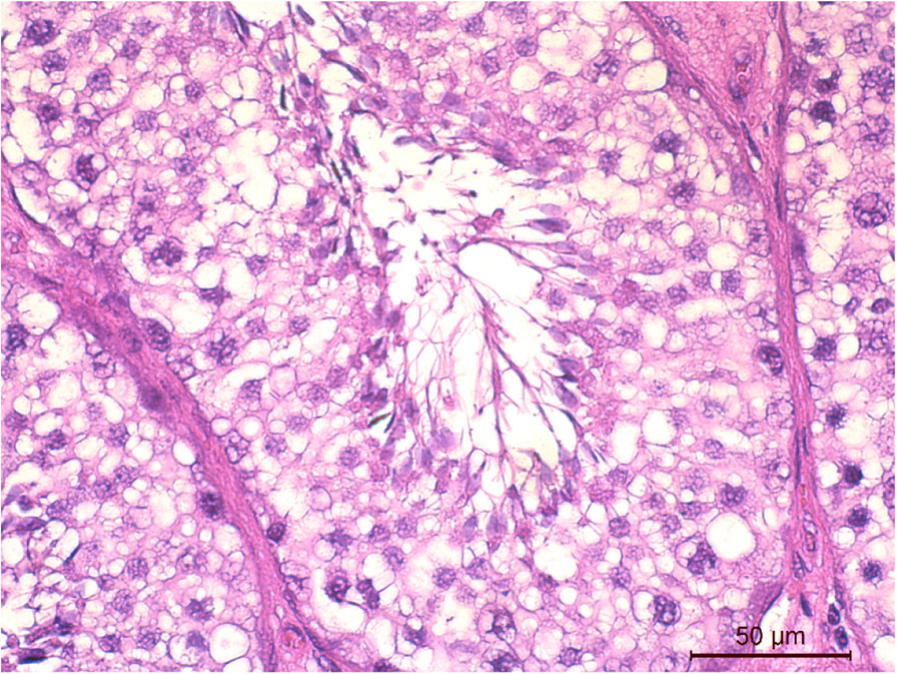
Histological evaluation of testis morphology from one buck of the altrenogest group (HE staining) A cross-section of a seminiferous tubule with elongated spermatids is shown

Histologically functional testicular tissue was detected in all animals (Fig. 3). Spermatozoa were present in the seminiferous tubule lumens except for in the intra-abdominal testicle of one experimental animal, in which no mature spermatozoa were detected.

## Discussion

The body sizes of the animals increased during the study. This can be explained by the fact that the goats were not fully grown at the beginning of the experiment. Treatment of male goats with altrenogest during the growth phase had no significant effect on the physical development of the animals, which is consistent with observations made in boars [9].

The time effect on the testicle volume in both groups is caused by the fact that testicle weight of seasonal bucks’ decreases with increasing daylight [14]. In the experimental group, the testes volume decreased more sharply during the experimental period, indicating an effect of the altrenogest application on the testicles. Although the changes in the testes sizes found in the present study were much smaller, this observation is consistent with the progressive decrease in testis size observed in boars and stallions after oral administration of altrenogest [9, 13]. The consistency was not significantly affected, arguing against an effect of 0.088 mg/kg altrenogest on goat testes. The absence of an effect of altrenogest on the testosterone concentration supports this assumption.

Statistical evaluation of the semen parameters must take into account the greatly varied response of the animals to the treatment, because the two experimental animals that produced good ejaculates make the overall results of both groups appear more similar than may be the case. A limitation of this study is the small number of treated animals, a greater amount of animals may have resulted in more significant results. The authors assume that a secure method for suppression of gonadal function requires an almost hundred per cent success rate. Against this background, the achieved results are meaningful despite the small sample size.

Particularly striking were the increased pathologic changes in the ejaculates of the experimental animals (Fig. 1). Head and tail anomalies of the spermatozoa were detected, which is consistent with the significant increase in head, tail, and midsectional abnormalities observed after altrenogest treatment in stallions [12, 13]. The mechanism by which altrenogest induces pathospermia requires further exploration. Unexplained cases of pathospermia in males may be linked to an increased endogenous progestogen concentration. In the previously mentioned studies on the stallion [12, 13], the observed treatment-induced changes were reversible. Similarly, in the present study, the pathologic changes in the ejaculate of the experimental animals diminished, although only at the end of the observation period.

The ultrasound findings in both the experimental and control animals were typical of adult goats [4, 15, 16]. Subsequent histologic examination revealed functional testicular tissue in all animals, confirming the reported correlation between the echotexture of the testes observed with ultrasonography and histologic appearance [17].

For both groups, there were no large fluctuations in the gray values. These findings indicate that the goats had already completed their adolescent development at the time the trial began; had the testicles not been fully developed, a clear increase in the gray scale would have been observed [17–19]. The gray scale values confirm the subjectively determined homogeneous echogenicity of the ultrasound images of both groups at all time-points.

Histologic examination revealed complete spermatogenesis in both testes of all animals at the end of the observation period. The only exception was in an intra-abdominal testicle of one goat; the histologic picture of this testis corresponded to the histologic appearance of cryptorchid testes [20].

## Conclusion

The present study evaluated the possibility of downregulating gonadal function in adult goats by the administration of altrenogest. Test animals exhibited significantly lower testicle volume and a significantly higher proportion of pathologically changed spermatozoa in the ejaculate; for all other parameters examined, no significant effects of treatment were detected. An individually different response of the goats to the treatment was observed especially with regard to testis consistency, libido scores, and occurrence of anejaculations. This could be due to differences in absorption and metabolism or insufficient dosage of the altrenogest. The observed effects were reversible. In summary, reliable suppression of reproductive function in the goat by once-daily administration of 0.088 mg/kg altrenogest was not successful. Further studies are required to determine the optimal dosage and application frequency, and the mechanisms leading to the induction of pathospermia.

## Methods

### Animals, characteristics, experimental setup

Eight Peacock goats (*Capra aegagrus hircus*, 8 months of age) were randomly divided into two groups and maintained under standard conditions. The Peacock goat, a medium sized goat, originated from Switzerland is used for the production of milk and meat. Sexual maturity occurs on average between 100 and 230 days of age. The Peacock goat is a seasonal breeder. The goats were kept in stables under natural light conditions. Before starting the experiment, the animals were accustomed to ejaculation using an artificial vagina, and the fertility and sexual maturity of all animals was verified by repeated sperm testing. One goat had a one-sided abdominal cryptorchidism, but because the animal had a normal sperm count and the ejaculate was of satisfactory quality, it was included in the analysis.

Over a trial period of 7 weeks starting on the 17th of December 2014, the four test animals were orally administered altrenogest (Virbagest 4 mg/ml oral solution for pigs; Virbac, France) once daily at the same time each day, and the four control animals were orally administered an equivalent volume of a 5% glucose solution. In each case, the amount administered was adapted to the current body weight of the animal. After completing the 7-week administration period, the goats were observed for additional 7 weeks to monitor the reversibility of the treatment’s effects on gonadal function.

### Recording of physical parameters, testicle measurements, and blood parameters

Every 14 days (days 1, 15, 29, and 43 of the experimental phase and days 57, 71, and 85 of the observation phase), the goats underwent clinical examination, including body weight, body length, and withers height; and testicle length, width, and depth (measured using a digital vernier caliper). Testicle volume was calculated according to the following formula which was used for bovine testicles [21]: testicle volume (cc) = 4/3 x π × 1/2 x testicle length × 1/2 x testicle width × 1/2 x testicle depth. Testicle consistency was assessed by palpation. A distinction was made between elastic (1), soft and elastic (2) and soft (3).

The examiner was not blinded to the treatment group of the goats.

A blood sample was collected from the jugular vein to check the plasma testosterone levels using a radioimmunoassay method with a lower detection limit of < 0.35 nmol/l, as described previously [22].

### Evaluation of semen parameters and behavior

At the above-described collection times, an artificial vagina was used to harvest ejaculate from the goats and the sperm were examined both macroscopically and microscopically [23, 24]. The ejaculate volume was measured, and the samples were assessed for color, consistency, odor, possible admixtures, and pH, and the density, mobility, and number were determined. After staining with bromophenol nigrosine, the proportions of live sperm and morphologically abnormal sperm were also determined. During the collection of each ejaculate, the reproductive behavior of the goats was scored according to a libido scale from 0 to 4 (Table 8).

**Table 8:** Libido classification of goats during the collection of ejaculates (modified after [25])

Score	Evaluation	Characterization of libido
0	No jump	No sexual interest on female goat
1	No ejaculation	Little sexual interest, no ejaculation within 20 min after first contact despite of several jumps and penetration of the artificial vagina
2	Substandard	Little sexual interest, up to 20 min between first contact and ejaculation
3	Standard	Moderate sexual interest, up to 5 min between first contact and ejaculation
4	Very good	Intense sexual interest, jump and ejaculation within one minute after first contact

### Ultrasound examination and gray value analysis

An ultrasound examination of the testes was performed at all time-points using the ultrasound unit HS 1500 V (Honda Electronics, Tokyo, Japan), a multi frequency microconvex transducer (model HCS-3710 M; 5.0/7.5/9.0 MHz) with a frequency of 7.5–9.0 MHz. Longitudinal and transverse sections of both testes were imaged with a linear probe and the homogeneity of the testicular tissue was monitored. To objectively quantify the ultrasonographic images, the images were subjected to a one-dimensional gray value analysis. For this purpose, two square regions of interest (ROI, 0.25 cm^2^) were selected in the testicular parenchyma (Fig. 2). For each pixel of the ROI, the intensity was scored between 0 and 255; the analysis was performed by the software of the ultrasound machine. To evaluate the gray value analysis, the mean gray value (Lmean) of both ROIs of each longitudinal and transverse section of the left and right testes was used. The mean of the eight gray values per animal at each time-point during the experimental and observation periods was determined. The detailed description of the method including the device settings is given elsewhere [26].

### Histologic examination

After completing the observation phase, the goats were castrated and the testicles were examined histologically. Two tissue samples were obtained from the parenchyma of each testicle, sectioned at a thickness of 3 μm, and stained with hematoxylin-eosin. The sections were then examined under a microscope for the presence of sperm cells.

### Statistical methods

The data were evaluated with the statistical programs BMDP/Dynamic, version 8.1 (Dixon, 1993) and R (version R3.2.3, FreeSoftware Foundation’s GNU project, 2015); Graphic illustrations were created using Microsoft Excel 2013 (Microsoft Corporation, Redmond, WA). First, the normality of the distribution of all parameters was evaluated using a residual analysis. For the normally distributed parameters, a two-factorial analysis of variance with repeated measures for each time-point was performed. Ordinal data were evaluated using a two-factorial approach, broken down into two one-factorial comparisons with non-parametric rank techniques. In addition, the Wilcoxon-Mann-Whitney test was used to test interactions between groups and time as well as the influence of the main effect group, and the Friedmann test was used to check the main effect time within groups. Results with a *p* value of ≤0.05 were considered statistically significant, with *p* ≤ 0.001 designated as a highly significant result.

## Data Availability

The datasets used and / or analyzed during the current study are available from the corresponding author on reasonable request.

## Abbreviations

L mean: Mean grey value
ROI: Regions of interest
